# Prognostic value of the C-reactive protein-albumin-lymphocyte index versus traditional inflammatory markers after radical resection of colorectal cancer: a retrospective cohort study

**DOI:** 10.3389/fmed.2025.1742517

**Published:** 2026-02-10

**Authors:** Zhicheng Jin, Mao Zhang, Yunqi Hua, Yuyang Deng, Shenghui Li

**Affiliations:** 1 Inner Mongolia Medical University, Affiliated Baotou Hospital of Inner Mongolia Medical University, Baotou, Inner Mongolia, China; 2 Department of Neoplasms Surgery, Baotou Tumor Hospital, Baotou, Inner Mongolia, China; 3 Baotou Tumor Hospital, Baotou, Inner Mongolia, China; 4 Baotou Medical College, Inner Mongolia University of Science and Technology, Baotou, Inner Mongolia, China

**Keywords:** colorectal cancer, C-reactive protein-albumin-lymphocyte index, inflammatory composite markers, LMR, NLR, PLR, prognosis

## Abstract

**Objective:**

To compare the prognostic value between the C-reactive protein-albumin-lymphocyte index (CALLY) and traditional inflammatory markers [including the neutrophil-to-lymphocyte ratio (NLR), the lymphocyte-to-monocyte ratio (LMR), and the platelet-to-lymphocyte ratio (PLR)] after radical resection of colorectal cancer (CRC).

**Methods:**

A total of 152 CRC patients who underwent radical resection in Baotou Central Hospital from January 2016 to December 2019 were selected and studied retrospectively. The clinicopathological traits of the patients were collected and analyzed, and their survival outcomes were followed up. The prognostic value of the CALLY index and classical CRC prognostic factors was compared through the concordance index (CI) and the area under the receiver operating characteristic curve (AUC). The COX risk regression model was used for multivariate analysis to evaluate the impact of different indicators on prognosis.

**Results:**

The AUC of the CALLY index was 0.789 (95%CI: 0.703–0.875, *P* < 0.001), which was significantly higher than that of NLR (0.664, 95%CI: 0.574–0.754), LMR (0.655, 95%CI: 0.559–0.751), and PLR (0.647, 95%CI: 0.553–0.740). The 5-year overall survival (OS) rate in the high CALLY group (≥ 1.045) was significantly better than that in the low CALLY group (83.5% vs. 12.9%, *P* < 0.001). Multivariate analysis showed that the CALLY index (HR = 0.124; 95%CI 0.060–0.255; *P* < 0.05) was an independent prognostic factor. Moreover, an increased CALLY index was associated with a better prognosis, suggesting this indicator is a protective factor of post-surgical prognosis in CRC patients.

**Conclusion:**

By integrating inflammation, nutrition, and immune status, the CALLY index performs significantly better than traditional single indicators in postoperative prognostic prediction in CRC patients. It can serve as a reliable tool for postoperative prognostic evaluation of CRC and provide incremental value for clinical risk stratification.

## 1 Introduction

Colorectal cancer (CRC) is a common malignancy worldwide, ranking among the top three most prevalent cancers globally, with the third-highest incidence rate and second-highest mortality rate (\1. Bray, Laversanne, Sung, Ferlay, Siegel, Soerjomataram <arttitle>Global cancer statistics 2022: globocan estimates of incidence and mortality worldwide for 36 cancers in 185 countries</arttitle>. <jrn>*CA Cancer J Clin*.</jrn>, 2024). In China, CRC incidence is second only to lung cancer, and it continues to rise annually, showing an increasing trend in younger individuals. As the global economy has developed, people are increasingly adopting unhealthy lifestyles and dietary habits, such as high fat intake, overeating, physical inactivity, and prolonged sitting, which have contributed to increased CRC incidence and mortality (2). CRC typically presents no obvious symptoms in its early stages, and most patients who do not undergo regular physical examinations often miss the optimal diagnostic window and timely treatment, being already in the intermediate or advanced stage when diagnosed. It’s typical clinical manifestations include changes in bowel habits, abnormal stool color and consistency, abdominal pain, abdominal masses, intestinal obstruction-related symptoms, and systemic symptoms such as anemia, weight loss, and fatigue (3). Currently, CRC treatment follows a surgery-based comprehensive approach, namely, radical resection supplemented by multidisciplinary team (MDT) strategies. The former is the preferred option for resectable tumors, and the latter includes chemoradiotherapy, targeted therapy, and immunotherapy (4). Despite advancements in surgical techniques and perioperative management, postoperative complications remain a critical factor influencing patient recovery and prognosis (5). Despite advancements in diagnostic and therapeutic techniques, postoperative complications continue to significantly impact patient recovery and prognosis. Accurate prognostic assessment constitutes a core component of clinical management for colorectal cancer (CRC). Appropriate prognostic markers not only enable precise stratification of patients into different risk categories to inform personalized treatment strategies but also effectively guide the optimization of postoperative follow-up protocols. This reduces the risks of overtreatment or undertreatment, thereby holding important implications for improving patient quality of life and conserving medical resources. Peripheral blood inflammatory markers have garnered significant attention in prognostic evaluation across various cancers due to their advantages of low cost, non-invasiveness, and easy clinical accessibility (6). Growing evidence indicates that these markers can not only predict the prognosis of cancer patients but also evaluate the efficacy of immunotherapy (7). However, current research on CRC still has significant limitations. On the one hand, the number of studies is limited. On the other hand, existing research mostly focuses on independent analysis of single or two inflammatory markers. Thus, there is no comprehensive multi-marker assessment system. To address this problem, researchers are making efforts to develop composite markers that integrate multiple inflammatory indicators to establish more accurate prognostic prediction models for CRC. The C-reactive protein-albumin-lymphocyte index (CALLY), first proposed by Japanese scholars in 2020, is a novel comprehensive indicator for assessing the prognosis of cancer patients. Studies have shown that it is an effective prognostic biomarker for post-surgical patients with hepatocellular carcinoma (HCC) (8–10). The CALLY index combines three key blood markers, namely, C-reactive protein (CRP), serum albumin, and lymphocyte count, which reflect a patient’s inflammatory status, nutritional condition, and immune function, respectively. While the CALLY index has been validated for prognostic prediction in various cancer patients, there remains a lack of systematic comparison between this index and traditional prognostic markers [such as neutrophil-to-lymphocyte ratio (NLR), lymphocyte-to-monocyte ratio (LMR), platelet-to-lymphocyte ratio (PLR), and carcinoembryonic antigen (CEA)] specifically in CRC patients. This study aims to compare the prognostic predictive performance of the CALLY index with that of traditional markers in CRC patients and assess its incremental value.

## 2 Materials and methods

### 2.1 General information

A retrospective analysis was performed on CRC patients who underwent radical resection at Baotou Central Hospital or Baotou Tumor Hospital from January 2016 to December 2019,The primary reasons for selecting this time period are as follows: ➀ To ensure an adequate follow-up duration (until December 31, 2024) for completing the 5-year survival outcome assessment; ➁ The hospital’s diagnosis and treatment procedures and laboratory testing methods were relatively standardized during this period, reducing the impact of technical variations on the results; ➂ The electronic medical record system had been fully established during this period, facilitating the extraction and verification of clinical data. According to the inclusion and exclusion criteria, a total of 152 patients were included in this study. (1) Inclusion criteria: ➀ Definite diagnosis of CRC (based on preoperative and postoperative pathological results). ➁ Blood tests performed within 1 week before surgery and no treatment promoting or reducing cell production (such as neoadjuvant radiochemotherapy). ➂ No complicated infectious diseases that could affect blood routine counts before surgery (such as lung and urinary infections). ➃ No metastasis to other parts detected by preprocedural examination. ➄ Complete clinical, pathological and follow-up data. (2) Exclusion criteria: ➀ Having other serious diseases that could affect survival before surgery, such as cardiopulmonary diseases. ➁ Having intestinal obstruction, intestinal perforation, or gastrointestinal hemorrhage requiring emergency surgery at the first admission. ➂ Being lost to follow-up or having incomplete medical records. ➃ Having other malignant tumors except CRC. This study was approved by the Ethical Review Committee of Baotou Central Hospital (No.: 2025-YJS 伦审-071), and the informed consent from patients was waived.

### 2.2 Statistical basis for sample size determination

In this study, the 5-year overall survival rate was used as the primary outcome measure, and sample size estimation methods related to survival analysis were employed. Referring to previous studies on prognostic markers in colorectal cancer, we assumed a 30% difference in 5-year survival rates between the high and low CALLY index groups. With a significance level set at α = 0.05 and a statistical power of 1-β = 80%, the minimum required sample size was calculated to be 120 cases using PASS 27.0 software. Considering the potential risks of loss to follow-up and data missingness inherent in retrospective studies, an additional 10% sample size redundancy was incorporated. Consequently, a total of 152 patients were ultimately included to ensure the statistical reliability of the study results.

### 2.3 Data collection and statistical indicators

The following patient data were collected through the electronic medical record system: ➀ Clinical data information: age, gender, body mass index (BMI), date of initial surgery, etc. ➁ Laboratory indicators within 1 week before surgery: neutrophil count, lymphocyte count, platelet count, monocyte count, albumin concentration, CRP, CEA, Carbohydrate antigen 199 (CA199), etc. ➂ Postoperative pathological data: tumor size, degree of differentiation, lymph node metastases (N stage), and tumor, node, metastasis (TNM) stage (8th edition), etc. ➃ Postoperative treatment information: chemotherapy regimen, number of chemotherapy cycles, and whether targeted therapy or immunotherapy is received. ➄ Statistical indicators: LMR = lymphocyte count (× 10^9^/L)/monocyte count (× 10^9^/L); NLR = neutrophil count (10^9^/L)/lymphocyte count (10^9^/L); PLR = platelet count (× 10^9^/L)/lymphocyte count (× 10^9^/L); CALLY = albumin concentration (g/L) × lymphocyte count (10^9^/L)/[CPR (mg/L) × 10]. All laboratory tests were performed in the Central Laboratory using standard methods.

### 2.4 Follow-up

The follow-up lasted from the day of surgery to December 31, 2024 and data analysis was made on a monthly basis. Information about patients’ survival, death, and disease progression was collected through outpatient visits, hospitalization re-examinations, and telephone consultations. The 5-year all-cause mortality, defined as the interval from the day of pathological diagnosis to the death of a patient caused by any reason during the follow-up period, was selected as the core observation indicator in this study. For cases that survived more than 5 years after diagnosis, their survival time was uniformly counted as 60 months. To ensure data accuracy, all data were independently verified by two researchers.

### 2.5 Statistical methods

Statistical analysis was performed using SPSS 27.0. The optimal cut-off values of preoperative LMR, NLR, PLR, and CALLY were determined using receiver operating characteristic (ROC) curves and the Youden’s index. Count data were expressed as n (%),Fisher’s exact test is used when the number of cases is < 5. Kaplan-Meier survival analysis was used to calculate patient survival rates, and the log-rank test was used for comparison. Univariate analysis was performed to compare survival rates among various groups. Indicators with statistical significance in univariate analysis were included in the COX proportional hazards regression model for subsequent multivariate analysis to evaluate the impact of different indicators on prognosis. The concordance index (CI) and the area under the ROC curve (AUC) were used to assess the prognostic value of the CALLY index and classic CRC prognostic factors among different groups. All results were considered statistically significant with *P* < 0.05.

## 3 Results

### 3.1 Relationship between the preoperative CALLY index and clinicopathological factors

Based on the maximal Youden’s index, the optimal cut-off value of the preoperative CALLY index was calculated as 1.045. Patients were divided by this optimal cut-off value into the low CALLY group (<1.045, *n* = 31) and the high CALLY group (≥ , *n* = 121). The clinicopathological traits between the low CALLY and high CALLY groups were compared. The results showed that there were no significant differences in age at diagnosis, gender, BMI, neoplasm location, neoplasm size, degree of differentiation, presence or absence of postoperative chemotherapy, CEA, and CA-199 between the low CALLY and high CALLY groups (*P*>0.05). The N and TNM stages showed significant differences between the low CALLY and high CALLY groups (*P*<0.05) (Table 1).

**TABLE 1:** Relationship between preoperative CALLY and clinical case factors.

Factor	<1.045 (*N* = 31)	=1.045 (*N* = 121)	χ^2^	*P*
Age			1.543	0.214
<65	11 (35.5)	58 (47.9)		
=65	20 (64.5)	63 (52.1)		
Gender			0.221	0.638
Male	17 (54.8)	72 (59.5)		
Female	14 (45.2)	49 (40.5)		
BMI			1.668	0.197
<24	30 (96.8)	108 (89.3)		
=24	1 (3.2)	13 (10.7)		
Tumor location			0.006	0.936
Left	20 (64.5)	79 (65.3)		
Right	11 (35.5)	42 (34.7)		
Tumor size			0.878	0.349
<3	2 (6.5)	15 (12.4)		
=3	29 (93.5)	106 (87.6)		
Lymph node metastasis			6.020	0.014
No	9 (29.0)	65 (53.7)		
Yes	22 (71.0)	56 (46.3)		
TNM			12.342	0.000
1+2	5 (16.1)	62 (51.2)		
3+4	26 (83.9)	59 (48.8)		
Degree of differentiation			0.322	0.851
Poorly	3 (9.7)	11 (9.1)		
Moderately	26 (83.9)	105 (86.8)		
Well	2 (6.5)	5 (4.1)		
Chemotherapy			0.662	0.416
No	6 (19.4)	32 (26.4)		
Yes	25 (80.6)	89 (73.6)		
CEA			4.316	0.038
<5	12 (38.7)	72 (59.5)		
=5	19 (61.3)	49 (40.5)		
CA199			15.890	0.000
<37	17 (54.8)	105 (86.8)		
=37	14 (45.2)	16 (13.2)		

### 3.2 Fisher’s exact test within groups under different TNM stages

The Fisher’s exact test was employed to analyze the prognostic impact of the CALLY index within groups stratified by different TNM stages. In TNM stage 1, the event rate was significantly higher in the low CALLY index group compared to the high CALLY index group (100% vs. 16.1%, *P* < 0.001). Similarly, in TNM stage 2, the event rate was also significantly higher in the low CALLY index group than in the high CALLY index group (84.6% vs. 16.9%, *P* < 0.001) (see Table 2).

**TABLE 2:** Fisher’s exact test within groups under different TNM stages.

TNM	CALLY	Total	Death	χ^2^	*P*
I+II	Low	5(7.5)	5(100)	–	<0.001
	High	62(92.5)	10(16.1)		
	Total	67	15(22.4)		
III+IV	Low	26(30.6)	22(84.6)	–	<0.001
	High	59(69.4)	10(16.9)		
	Total	85	32(37.6)		

### 3.3 Relationship between the CALLY index and the survival of CRC patients

**FIGURE 1:**
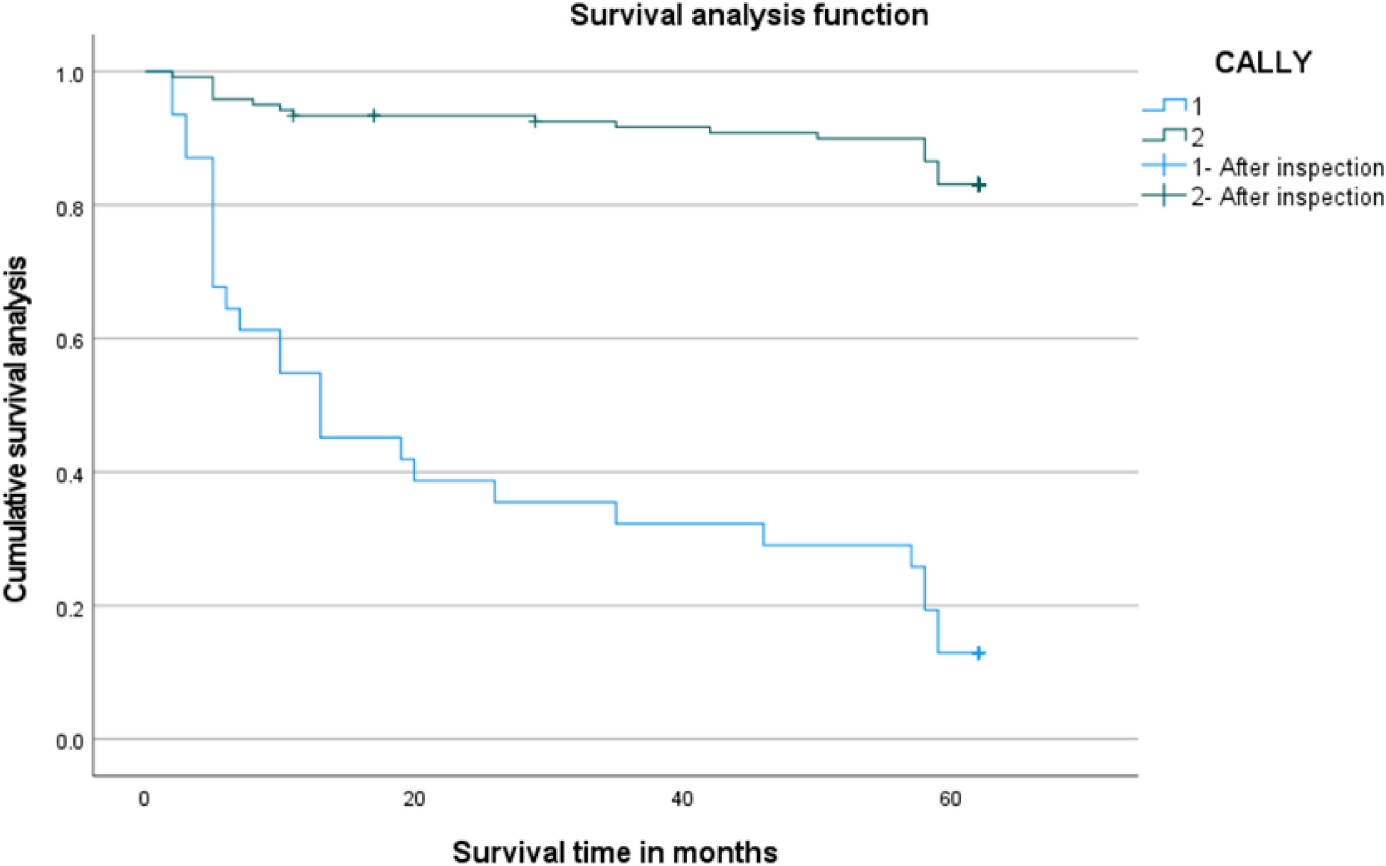
Survival analysis of CALLY groups. The two curves represent the high CALLY group (≥ 1.045, *n* = 121), and the single curve represents the low CALLY group (< 1.045, *n* = 31), *P* < 0.001.

Kaplan-Meier survival analysis was used to calculate the survival rates of patients, and the log-rank test was employed for intergroup comparisons to evaluate prognostic factors in CRC patients. Univariate analysis showed that patients in the low CALLY group (< 1.045) had a significantly lower 5-year OS rate than those in the high CALLY group (≥ 1.045) (12.90% vs. 83.50%, *P* < 0.001). Multivariate analysis using the COX regression model revealed that preoperative high CALLY levels (≥ 1.045) reduced the mortality risk by 87.60% in CRC patients, compared with low CALLY levels (< 1.045) (HR = 0.124, 95%CI: 0.060–0.255, *P* < 0.001) (Figure 1).

### 3.4 Prognostic value of the CALLY index and classic CRC prognostic markers

**FIGURE 2:**
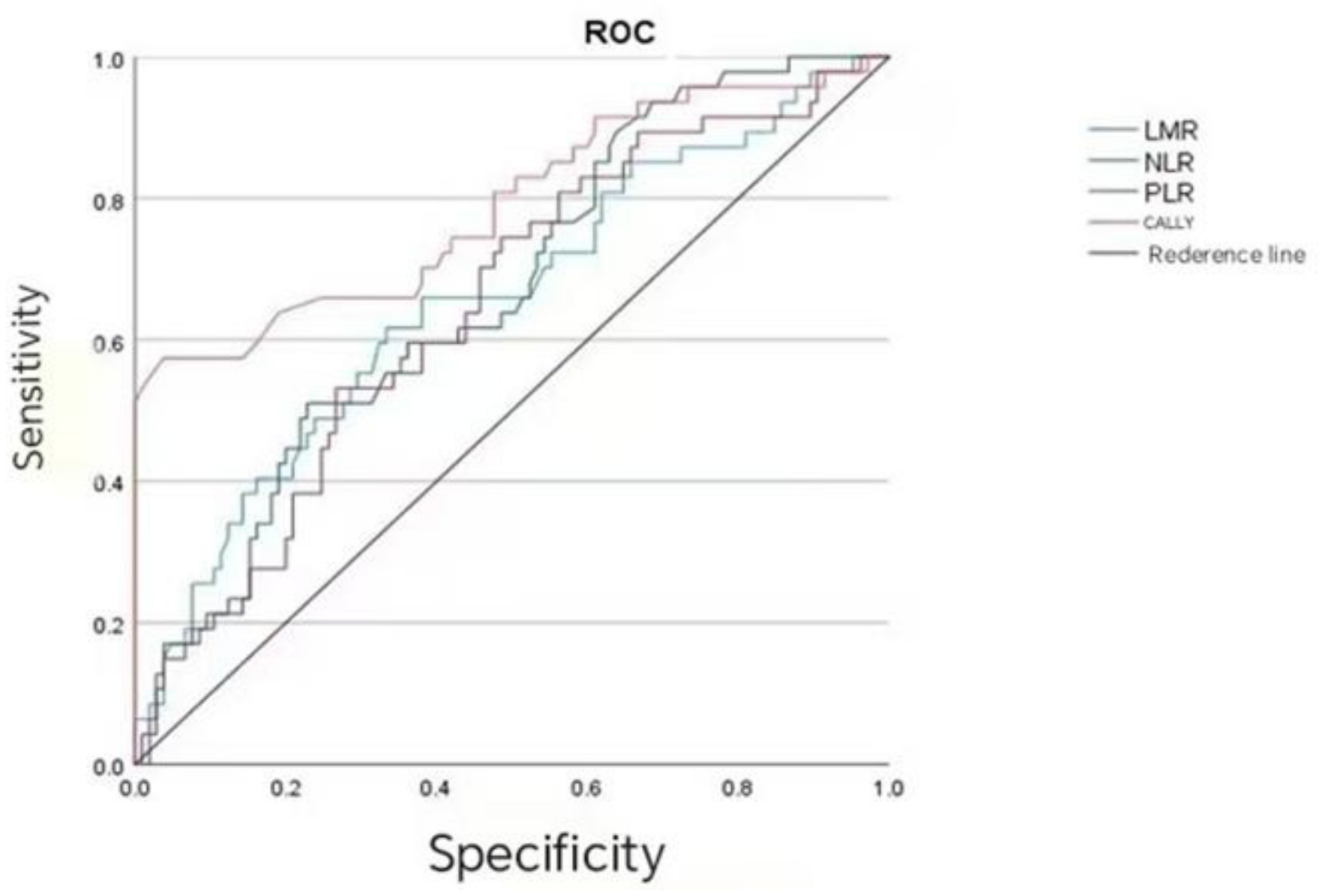
Analysis of the efficacy of CALLY, LMR, NLR, and PLR in evaluating the prognosis of CRC patients.

As shown in Table 3, the prognostic value of the CALLY index (AUC = 0.789, 95%CI: 0.703–0.875, *P* < 0.001), NLR (AUC = 0.664, 95%CI: 0.574–0.754, *P* = 0.001), LMR (AUC = 0.655, 95%CI: 0.559–0.751, *P* = 0.002), and PLR (AUC = 0.647, 95%CI: 0.553–0.740, *P* = 0.004) was analyzed. The CALLY index showed the highest prognostic value for CRC patients, followed by NLR, LMR, and PLR successively. The results of the COX regression analysis revealed that the *P*-value of CALLY grouping was < 0.001, while no significant difference was observed in *P*-value among LMR grouping (0.699), NLR grouping (0.498), and PLR grouping (0.367) (Figure 2 and Table 4).

**TABLE 3:** Critical values and AUCs of preoperative LMR, NLR, PLR, and CALLY.

Index	AUC	95%CI	Sensitivity	Specificity	Youden’s index	Critical value	*P*
LMR	0.655	0.559–0.751	0.617	0.667	0.284	2.925	0.002
NLR	0.664	0.574–0.754	0.511	0.771	0.282	2.855	0.001
PLR	0.647	0.553–0.740	0.745	0.514	0.259	151.47	0.004
CALLY	0.789	0.703–0.875	0.574	0.962	0.536	1.045	0.000

LMR, Lymphocyte-to-monocyte ratio; NLR, Neutrophil-to-lymphocyte ratio; PLR, Platelet-to-lymphocyte ratio; CALLY, C-reactive protein-albumin-lymphocyte index.

**TABLE 4:** Survival COX regression.

Variable	B	SE	Wald	*P*	HR	95%CI
LMR grouping	−0.142	0.366	0.150	0.699	0.868	0.424–1.778
NLR grouping	0.258	0.380	0.460	0.498	1.294	0.614–2.728
PLR grouping	0.373	0.414	0.812	0.367	1.452	0.645–3.268
CALLY grouping	−2.089	0.368	32.175	0.000	0.124	0.060–0.255

## 4 Discussion

In recent years, identifying predictive biomarkers, particularly those closely linked to inflammatory responses and tumor biological characteristics, has emerged as a focus in cancer prognosis research. Historically, researchers primarily analyzed single inflammatory markers or pairwise combinations such as LMR, NLR, and PLR. As research advances, the CALLY index—a composite of multiple markers—has gained increasing attention for its ability to more comprehensively capture the complex interplay between inflammation and tumors. Multiple studies have proven its significant association with poor prognosis in cancer patients (11, 12). However, there remains a paucity of CRC-specific research, and controversies persist regarding the prognostic impact of various inflammatory markers in CRC patients. This situation highlights the need for more targeted research to clarify its clinical value.

As early as 1,863, Rodolph Virchow observed white blood cells in tumor tissues and first proposed the hypothesis that the development and progression of malignant tumors are linked to inflammation. Subsequent advances in research have enhanced our understanding of the tumor microenvironment (TME), which supports his initial hypothesis. CRPs are an acute-phase reactant whose elevated levels indicate systemic inflammation, and previous studies have confirmed abnormal increases in CRP levels among cancer patients (13). Malnutrition is a common complication of malignant tumors. Some deaths are even attributed to malnutrition rather than the cancer itself. Serum albumin is a key indicator of nutritional status (14, 15). Lymphocytes are the core effector cells of anti-tumor immunity. A reduced lymphocyte count impairs the body’s adaptive immune function and weakens its capacity for immune surveillance and to eliminate tumor cells (16). The interplay among these three factors is complex: Inflammation accelerates nutrient depletion and compromises immune function, while malnutrition not only impairs immune responses but also elevates inflammatory levels through intestinal microbiota (17). A lower CALLY index indicates a robust inflammatory response coupled with poor nutritional and immune status, presenting a negative correlation with prognosis. Data from this study showed that the 5-year OS rate was significantly lower in the low CALLY group (< 1.045) than that in the high CALLY group (≥ 1.045) (83.50% vs. 12.90%, *P* < 0.001). It should be noted that 1.045 was the optimal cut-off value of CALLY calculated via the Youden’s index. Multivariate analysis via COX regression modeling revealed that patients with high preoperative CALLY levels (≥ 1.045) exhibited an 87.60% reduction in mortality risk compared with those with low CALLY levels (< 1.045) (HR = 0.124, 95%CI: 0.060–0.255, *P* < 0.001). Previous research (18) has demonstrated that the CALLY index correlates strongly with N and TNM staging in CRC patients, which is consistent with our findings. Similarly, Takeda et al. (19, 20) identified the CALLY index as a potential independent prognostic biomarker for long-term outcomes in CRC patients. Aligning with our results, both studies confirmed a negative correlation between the CALLY index and CRC prognosis, establishing it as an independent prognostic factor. This finding is consistent with prior observations in gastrointestinal malignancies including esophageal cancer (21) and pancreatic cancer (22). Building on the above-mentioned findings, the CALLY index developed in this study integrates three representative biomarkers: inflammation (CRP), nutrition (serum albumin), and immunity (lymphocytes). It not only leverages their independent prognostic value but also comprehensively predicts outcomes in CRC patients by capturing their interactions (23, 24).

In the present study, the prognostic predictive efficacy was systematically compared between the CALLY index and classical inflammation markers. The results showed that the AUC of the CALLY index reached 0.789 (95%CI: 0.703–0.875), which was significantly higher than that of LMR (0.655), NLR (0.664), and PLR (0.647). A closer AUC to 1 often indicates stronger ability to distinguish prognostic outcomes (such as relapse and survival difference). A larger CALLY index means that it can more accurately identify the prognostic risk in CRC patients, which is consistent with the research results of Yang et al. (25). It indicates that the CALLY index is superior to classical CRC prognostic markers (NLR, LMR, and PLR) in prognostic efficacy. The results of COX regression analysis showed the *P*-values of these three classical CRC prognostic markers were > 0.05, which suggests they have no association with prognosis.

A recent study published in Medicine reported that a higher preoperative lymphocyte-to-monocyte ratio (LMR) independently predicted improved overall and recurrence-free survival in patients with recurrent colorectal cancer (26). This finding parallels the core premise of our study, as both analyses underscore the prognostic significance of systemic immune-inflammatory balance in colorectal cancer. The shared observation that preoperative inflammatory markers—whether composite indices such as CALLY or individual markers such as LMR—can stratify patients according to survival risk highlights the broader value of inflammation-based biomarkers in guiding postoperative surveillance and clinical decision-making. Integrating evidence from these independent cohorts strengthens the rationale for further prospective investigations and supports the potential clinical utility of such markers in personalized management of CRC (27).

## 5 Conclusion

This study has several limitations. First, as a retrospective investigation, it cannot eliminate selection bias associated with patient backgrounds, which may compromise the objectivity of the results. Second, the study included only 152 CRC patients, resulting in a relatively small sample size that might impact the statistical power of the findings. This issue is particularly evident in subgroup analyses, where certain subgroups contained even fewer casesg in aas only 19 patients with right-sided CRC in the low CALLY groupned even fewer casesg in a reltability of the results. Third, there currently exists no consensus regarding the standardized cutoff value for CALLY or its clinical application. The cutoff value of 1.045 identified in this study has not undergone external validation, limiting its clinical utility. Future multicenter studies with larger sample sizes will be necessary to validate this threshold. Fourth, the details of postoperative treatments (such as chemotherapy regimens, cycles of chemotherapy, and receipt of targeted therapy or immunotherapy) and their potential roles as confounding factors have not been sufficiently reported. In this study, only whether patients received postoperative chemotherapy was recorded, while detailed information including specific chemotherapy regimens (e.g., FOLFOX, CAPOX), number of chemotherapy cycles, and treatment adherence was not collected. These factors may influence patient prognosis and thereby interfere with the correlation analysis between the CALLY index and prognosis. Fifth, this single-center study primarily enrolled participants from the Inner Mongolia region, which may introduce geographical limitations and restrict the generalizability of the results.

Based on the findings of this study, we propose the following clinical recommendations: the CALLY index could serve as an auxiliary tool for postoperative prognostic evaluation in CRC patients. Patients with a preoperative CALLY index < 1.045 may belong to a high-risk population, for whom enhanced postoperative follow-up monitoring (e.g., shortened follow-up intervals, increased frequency of imaging examinations and tumor marker detection) and consideration of more aggressive adjuvant treatment strategies (e.g., prolonged chemotherapy cycles or combination with targeted therapy) are recommended. Additionally, for patients with a lower CALLY index, emphasis should be placed on perioperative nutritional support and anti-inflammatory therapy to improve their nutritional status and immune function, thereby enhancing prognosis. Future research should involve prospective, multicenter studies with large sample sizes to further validate the prognostic value and optimal cutoff value of the CALLY index, as well as to explore its application in guiding individualized treatment strategies.

## Data Availability

The original contributions presented in this study are included in this article/supplementary material, further inquiries can be directed to the corresponding author.
